# The Editorial Function

**Published:** 1894-10

**Authors:** Henry Burchard

**Affiliations:** Philadelphia


### THE EDITORIAL FUNCTION.

BY HENRY BURCHARD, M.D., D.D.S., PHILADELPHIA.

How many of us appreciate in a full degree the importance—the vital importance—of an editor's work? How many recognize the true part he plays in matters relating to our profession?—that he is not a machine for punctuating and paragraphing essays, but a living, active force in whatever work he may be engaged?

Some forty or fifty years ago, when dentistry was in a chaotic, or, at least, an inchoate condition; when dental practitioners of the better type were extracting from the half-concealed charlatanism of dental carpenters the material from which to build a profession, editing might and could have been viewed in a careless or an uncritical manner. The field then covered called but for slight ability to meet all needs.

With rare exceptions, the material furnished for discussion was mainly speculative, tentative thinking on the way to science, so that much latitude could be taken and allowed as to the acceptance or rejection of articles.

Since then the growth has been constant, a steady advance, until now the pseudo-scientific is relegated to its proper position and the scientific receives endorsement.

Editors are popularly and truly supposed to be the educators of their readers, this not alone through their own literary work, but by the material selected for the readers' perusal.

It is not necessary to state explicitly, as is done in the Chautauqua text-books, that the editor or journal is not responsible for, nor does he endorse, all the views and opinions enunciated by a contributor. However, the acceptance and publishing of articles is endorsement in some measure; he may not accept as truth, or be persuaded by all he publishes, still the fact of publishing is a tacit recognition that the printed article is worthy of the attention of readers. This may seem to have no direct bearing upon the practice of dentistry. Perhaps it has not; but it is of vital importance to the science of dentistry.

Complaints are general that many contributors to the journals, and many of those who take part in the discussion of essays in society meetings, constantly ignore the general principles upon which dental science is based. So many work and reason without due recognition of the principles of general science, that the results of their work become well-nigh invalid. Many fine-spun theories and bold hypotheses, seemingly fair to the casual glance, are, by a species of *reductio ad absurdam*, resolved into self-contradiction or drivelling puerilities.

We know, of course, that the cure of this state does not lie with the editor, and presumably it is a matter of regret that this should be true; but, fortunately, he has in his grasp, if not the reversing apparatus, the brakes to apply.

It is the use of these brakes which distinguishes the editor of mind from the editor of the scissors. The word editor is applied, of course, to the man who discriminates as to the worth of unpublished matter. He who clips the already-printed is not an editor, but a journalistic tailor; it is steel shears in contradistinction to brains.

One naturally expects as a first characteristic in an editor that he possess what is known as the judicial cast of mind,—that is, the capability of comparing and weighing statements and evidence presented to him.

In one meeting a half-dozen conflicting sets of views may be presented, and in that meeting no balance is struck; each advocate carries with him a confident belief that his views are the correct ones. It is here the well-equipped editor plays his part. From the tangled maze of controversy he unravels the many lines, lays them in a row, and measures their relative worths. The writer once stated in an essay "that dentistry had no court of appeals." The recantation is here: the court exists in the sound professional editor, for he weighs after the evidence is all in.

As every man's baby is finer than his neighbor's, and doubly outweighs the offspring of his antagonist, each stands to his own opinions. The fact of a man having a bias warps his judgment; so the sound and rational opinion must come from one who has no pet child to coddle.

The editor has before him in panorama the pictures of his immediate science and those collateral; the chance writer has dangling before him an edge of one of these pictures, sees but that fragment, and how can he think beyond it; and how can an editor see but in his field of vision. All men have an intellectual horizon; according to the man, this may be six inches, six miles, or in some instances be well-nigh boundless. It is for breadth of horizon that editors should be, and presumably are, selected. Though it is important that each learn exhaustively all that lies in his particular circle, it is more important that this annular journey should have as large a circumference as is consistent with easy grasp.

At the extremes we have the data-hunter and the generalizer—the microscopist *per se* and the cosmologist. To which of these does one turn when he desires formulated knowledge. To one the $\frac{1}{15}''$ objective, the chemical-balance spectroscope or telescope is a means to an end, an instrument, an incidental in the reasoning to principles. To the other the field of his objective is his horizon, the spectrum the only arrangement of colors; all the universe may be weighed in his scales, and these instruments make his world.

Who can deny that, although it is a requisite to have fact-findders, phenomena-recorders, and data-searchers, it is equally important that we have the results of their work generalized and systematized; to have an impartial viewer blow, and blow hard, the wheat from the chaff; for to give us wheat alone is the editorial function.

